# Regulation of the Colour Change of 3D-Printed Mackerel Mince (*Scomber scombrus*) Based on Purple Potato Powder and Citric Acid

**DOI:** 10.3390/foods12061342

**Published:** 2023-03-22

**Authors:** Zheng Jin, Yisha Xie, Zheming Wang, Yue Wang, Qinxiu Sun, Xiuping Dong

**Affiliations:** 1 National Engineering Research Center of Seafood, Collaborative Innovation Center of Seafood Deep Processing, Liaoning Province Collaborative Innovation Center for Marine Food Deep Processing, School of Food Science and Technology, Dalian Polytechnic University, Dalian 116034, China; 2 School of Food and Bioengineering, Xihua University, Chengdu 610039, China; 3 College of Food Science and Technology, Guangdong Ocean University, Zhanjiang 524088, China

**Keywords:** mackerel, purple potato powder, citric acid, colour, 3D printability

## Abstract

The present study evaluates the effect of purple potato (PP) powder and citric acid (CA) on the regulation of the colour change of 3D (three-dimensional) printed mackerel mince (*Scomber scombrus*). In addition, the effects of PP and CA content on the 3D-printability and quality of mackerel mince were also investigated. The results showed that an increase in PP and CA concentrations gradually brightened the product colour and turned it pink. Furthermore, an increase in PP concentration and added CA reduced the fluidity and loss of water in mackerel mince. Proper PP and CA concentrations moderately increased the storage modulus (*G*′), loss modulus (*G*″), and yield stress of mackerel mince, making it suitable for 3D printing. At the same time, an increase in PP and CA concentrations enhanced the umami and sweet taste of mackerel mince but reduced the fishy and sour taste, and the degree of preference was within the acceptable range, except for PP1%-CA0%. It was found that, when the 3D-printing accuracy of mackerel-mince samples reached more than 97% and was acceptable, the optimal PP and CA concentrations for realizing the regulation of L*, a*, and b* were 1.00~3.00% and 0.09~0.32%, respectively.

## 1. Introduction

Atlantic mackerel (*Scomber scombrus*) fits in the class of economical seafood with high nutritional value [1], low price, and worldwide distribution. It is often industrially processed into canned and cured products, including dried fish fillets [2], preserved fish, smoked products, and minced fish products, or directly frozen for consumer consumption [3]. Compared to other fish products, the variety of mackerel products is still low, and there is an urgent need to research new processing processes and technologies for mackerel products [4]. Food 3D (three-dimensional) printing is a new type of intelligent molding technology that has unique properties in terms of convenience, flexibility, efficiency, and tailored nutrition [5]. Minced mackerel is a soluble gel with certain viscosity and fluidity, which can form a gel with elasticity, and from the raw material analysis, it is a suitable food material for 3D printing [6]. Therefore, combining 3D printing with mackerel surimi can develop a convenient, ready-to-eat food with controllable nutrition content and customized shapes. However, minced mackerel has the problems of dark colour and poor taste [7], which affect people’s appetite and taste. Therefore, it is necessary to add appropriate ingredients to the minced mackerel to improve the colour of the fish paste. Purple potato powder is rich in bioactive and functional compounds such as anthocyanins [8], which can give mackerel puree an attractive colour and increase people’s appetite. It has been shown that adding sweet potato starch to food ingredients could give them an attractive colour [9,10] and improve the 3D printability of food [11]. However, it still has the problem of being singular in colour and easily faded after heat treatment. The anthocyanins in purple potato (PP) are degraded by temperature, oxygen, and pH during processing, resulting in poor colour. Citric acid (CA) is widely used as a colour preservative and acidity regulator in the meat industry, and it is generally considered safe by the Food and Drug Administration (FDA) [12]. The addition of CA to purple potato puree can chelate metal ions to protect the colour of anthocyanins, while the addition of different levels of CA can also achieve a colour regulation of PP by adjusting the pH value. However, excessive CA addition will reduce the pH of fish products, resulting in gelling problems, which will affect the taste and texture of the fish [13].

With the rapid development of science and technology, people are more eager to find a quick and easy cooking method to meet their fast-paced lifestyles. Microwave baking stands out for its convenience, speed, and hygiene, while saving time and energy. Due to the short heating time, the nutrients in the food can be more retained and the water loss in the product is greatly reduced, thus ensuring the flavor and taste of the food.

Therefore, the effects of different amounts of PP and CA on the colour regulation of 3D-printed mackerel-mince microwave products were studied in the present study, and these provided a reference for the development of mackerel snacks. Since the research subject is a 3D-printed product, the premise of the experiment is whether the raw material can be printed and what the effects of PP and CA content on the 3D printability, rheological properties, texture properties, moisture properties, and sensory qualities of 3D-printed mackerel mince. In the correlation analysis, a highly correlated index, sensory index, and colour were used as predictive values, and the process prediction model was established with PP and CA to accurately obtain the optimal PP concentration, CA added amount, and colour range, to obtain popular products with a good 3D-printing effect.

## 2. Materials and Methods

### 2.1. Materials

Frozen Atlantic mackerel (*Scomber scombrus*) was purchased from Dalian Fresh Market (Dalian, China), transported to the laboratory in a −20 °C refrigerated truck, and stored in a −20 °C refrigerator. Tea polyphenols (TP) (food grade) were purchased from Henan Wanbang Chemical Technology Co., Ltd. (Henan, China). PP powder (food grade) was purchased from Yunnan Yi Xiao Bake Trading Co., Ltd. (Yunan, China). CA (food grade) was purchased from Zhejiang Yin Biotechnology Co., Ltd. (Zhejiang, China). Salt, cooking wine, and sugar were all food-grade and were purchased from Dalian Qianhe Market (Dalian, China). Ultra-pure water was used throughout the experimental process.

### 2.2. Experimental Design

**Figure 1:**
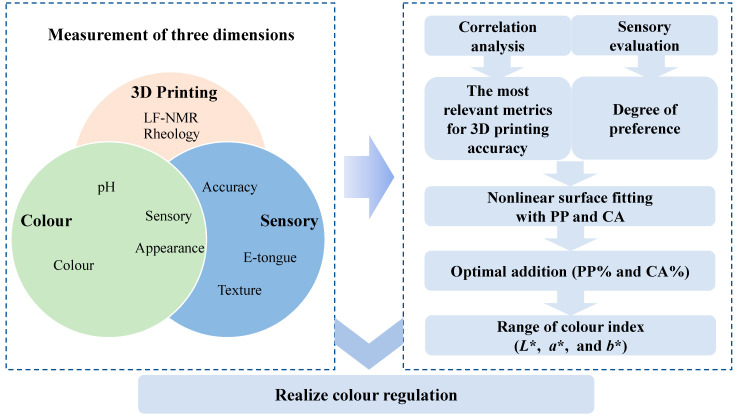
Experimental considerations.

The aim was to investigate and achieve the colour regulation of mackerel mince from three dimensions (3D printability, colour, and sensory). Dimension one was 3D printability, measuring rheological, low-field nuclear magnetic resonance (LF-NMR), 3D printing accuracy, and pH indices of products, while observing appearance/morphology (Figure 1). The acidity of CA changes the colour of PP, hence, pH can also express the colour dimension of mackerel mince indirectly. Finally, the printing accuracy and appearance/morphology were combined with electronic-tongue, texture, and sensory evaluations to form the sensory dimension. Among them, 3D printability was the most important characteristic, so all indicators of the 3D printing dimension were subjected to a correlation analysis. In the correlation analysis, the two indexes most relevant to 3D-printing accuracy were selected. The 3D-printing index and sensory index were fitted with the PP concentration and CA amount added. The optimal amounts of PP and CA added were obtained through the range of sensory acceptability (>5) and the range of printing accuracy (>97%). Similarly, the colour index was fitted with the PP concentration and CA amount to obtain the colour range of mackerel mince under the optimal amount added.

### 2.3. Preparation of Minced Fish

The frozen mackerel was thawed in the refrigerator (4 °C) until the central temperature reached −2 °C. After the head, tail, skin, and gut of mackerel were removed, we took 100 g mackerel flesh (13% of dark meat, 87% of white meat, and 0.2 g/kg TP) and chopped it in a meat grinder (QSJ-D03Q1, Bear, Guangdong, China) for 5 min. The first chopping step was to make the dark meat and white meat mix more evenly. The mixture was then passed through a 40-mesh sieve to obtain a pure mackerel-mince sample.

Moreover, He et al. [9] found that a 3% PP solution (30 g PP powder mixed with 100 mL of boiling water) also had good performance for 3D printing alone. In addition, mackerel mince had certain viscosity, so 3%PP was taken as the maximum value and reduced to 2% and 1%. The amount of CA added was determined using sensory pre-experiments, and it was found that the maximum amount of CA that people could accept was 0.6%. When the CA content was greater than 0.6%, the 3D printing effect was not good. Therefore, CA peaked at 0.6% and then decreased to 0.3% and 0%. According to the above thought, the pure mackerel mince was further processed.

The ingredients included sugar (9%), salt (2%), cooking wine (2%), pure mackerel mince (77%, 76.7%, and 76.4%, mass ratio(*w*/*w*)), with a fixed addition of 10% PP solution of different concentrations (the concentrations of PP solutions were 1%, 2%, and 3%, and the configuration method was 10 g, 20 g, and 30 g PP power dissolved in 100 mL boiling water, respectively.) and CA of different amounts added (0%, 0.3%, 0.6%, respectively). Then the mixture was chopped for 100 s to obtain samples. The purpose of chopping this time was to make the mixture of pure mackerel mince and compound more even. Finally, the product was processed by microwave (340 W, 5 min).

The PP concentration, CA amount, and the names of each treatment group are shown in Table 1. Rheological properties of mackerel mince were measured before printing. After 3D printing, the sample was analyzed for texture, colour, printing performance, and calculated printing accuracy, and then after heat treatment, the sample was analyzed according to appearance, texture, sensory expression, colour, and electronic tongue.

According to the previous studies of the same research group [11], adding potato starch made mackerel have good printing performance. This was because the test group without PP added was not set when different concentrations of PP were investigated in this study.

### 2.4. Measurements of Colour

The colour (including L*, a*, and b* values, which are, respectively, brightness value, red and blue value, and yellow and green value) of the 3D-printed mackerel mince sample before and after microwave-heat treatment were measured using a colourimeter (SC-80C, Kyowa Konko, Beijing, China) according to the method of Lin et al. [3]. Each sample was run three times in parallel.

### 2.5. Measurements of Rheological Properties

The rheological properties were measured with a rheometer (Rheometrics Discovery HR-1, TA Instruments, New Castle, DE, USA) according to the method of Xie et al. [14] with minor modifications. Measurements were performed using a parallel-plate geometry (diameter, 20 mm, gap, 1 mm). Samples were left on the lower plate for 60 s to ensure thermal and mechanical balance. The temperature of the Peltier apparatus was controlled at 25 ± 0.1 °C.

A dynamic strain scan was performed to determine the linear viscoelastic region (LVR) in the strain range of 0.01–100%. Frequency scan tests were performed for the same strains (20%) in the LVR frequency range of 0.1–100 rad/s [15,16]. The shear stress and viscosity of each sample were recorded.

The determination of yield stress was conducted according to the method of He et al. [10], and the storage modulus (*G*′) and loss modulus (*G*″) were obtained at an angular frequency of 1 to 3000 Pa with a fixed frequency of 10 rad/s. The yield stress was defined as the intersection of the storage modulus and loss modulus.

### 2.6. 3D Printing

The mackerel mince was printed with an extrusion-type food 3D printer (FOOD-B2, Changxing Shiyin Technology Co., Ltd., Zhejiang, China) at 4 °C. To facilitate the measurement of 3D printability, the print geometry was designed as a square (42 mm side length, 4 mm height, 6 layers). The printing parameters were set as follows: the nozzle diameter was 0.84 mm, layer height was 1.0 mm, movement speed was 20 mm/s, extrusion force was 5–7 N, filling density was 80%, and fill mode was linear filling.

### 2.7. Analysis of Printing and Deformation

Vernier calipers were used to measure the center height and edge length of the 3D printed samples before and after microwave heat treatment. The suitability of the material for 3D printing was judged by the difference between the final measured edge lengths and heights and the model parameters. The printing accuracy was calculated referring to Pan et al. [17].

(1) $$\mathrm{PA}\;(\%)=(1-\frac{|x_{1}-42|}{42})\times 100,$$

(2) $$\mathrm{DD}\;(\%)=\frac{\left|x_{2}{-x}_{3}\right|}{x_{2}}\times 100,$$

PA is the printing accuracy of 3D printing products, %; DD is the degree of deformation of 3D printing products, %; *x*_1_ is the 3D printing products’ side length, mm; *x*_2_ is the 3D printing products’ side length or height, mm; *x*_3_ is the thermal products’ side length or height, mm; 42 is the 3D print geometry’s side length, mm.

### 2.8. Measurements of pH

The pH of 3D-printed mackerel-mince samples before and after microwave heat treatment was measured with a pH meter (FE28/38, METTLER TOLEDO, Columbus, OH, USA). Briefly, 2 g of the sample before and after microwave heat treatment was added to 10 times (*w*/*v*) deionized water, homogenized (T18, IKA, Staufen, Germany) for 15 s, and then centrifuged with a centrifuge (CF16RN, HITACHI, Tokyo, Japan) at 4 °C, 5000 r/min for 10 min, and the supernatant was used to measure the pH. This method was used to determine the pH of seasoning to be 7.09, which has no significant effect on the pH of prepared mackerel mince.

### 2.9. Measurements of Crispness and Hardness

The determination of texture properties of the heat-treated samples was conducted according to the method described by Abraha et al. [18] with minor modifications to the test speed and puncture distance. After heat treatment, the sample was cooled to room temperature. The heat-treated samples were subjected to crispness analysis using a physical property analyzer (TA-XT Plus, Stable Micro Systems, God-alming, UK). The moment arm is 30 kg, the *P*_2_ probe (diameter 2 mm) was selected, the puncture distance was 30mm, the pre-test and post-test speeds were 1.00 mm/s, and the test speed was 5.00 mm/s. Room temperature (approximately 25 °C) was maintained during the test.

### 2.10. LF-NMR Analysis

A low-field NMR analyzer (NMI20-030H-I, Suzhou Niumai Analytical Instrument Co., Suzhou, China) was used to determine the migration of water molecules in the 3D-printed sample according to the method of Melis et al. [19]. First, a homogeneous field correction process was performed, the standard solution was placed in the center of a 40 mm diameter RF coil, and the Q-FID mode was selected for correction to obtain the center frequency and pulse time. Subsequently, the determination of sample moisture migration was performed by placing the sample to be measured in a 40 mm diameter test tube and inserting it into a magnetic field with an intensity of 0.5 T and a frequency of 21 MHz. The lengths of the two pulses were set to 31.0 µs and 62.0 µs, respectively, and the transverse relaxation time (*T*_2_) was measured using the Carr-Purcell-Meiboom-Gill (CPMG) sequence. The relaxation intensity was obtained by one-component inversion and the relative peak area (*P*_2_) was obtained by calculation.

### 2.11. Electronic Tongue Analysis

The five basic taste attributes (sour, salty, sweet, bitter, and fresh) and four combined attributes (richness, umami, aftertaste-A, aftertaste-B, and astringency) of the samples after heat treatment were measured by electronic tongue (TS-5000Z, INSENT, Kanagawa Prefecture, Japan) according to the method of Zhou et al. [20] with slight modifications. We precisely weighed 2 g of the sample in a 50 mL centrifuge tube with 25 mL of deionized water, homogenized it for 10 s, and then let it stand for 10 min. The mixture was then centrifuged at 8000 r/min for 10 min at 4 °C using a centrifuge (CF16RN, Hitachi, Tokyo, Japan), the supernatant was filtered, and the volume was fixed to 100 mL with deionized water. A sample required two sample cups, each of which was filled with 40 mL of sample solution.

Electronic tongue parameters were set as follows: the equilibrium solution was deionized water, sample volume was 80 mL, analysis time was 180 s, acquisition time was 120 s, the sensor collected one piece of data per second, the response value of 120 s was selected as the original data signal for analysis, rinsing time was 10 s, sample temperature was 20 °C, each sample was repeated 5 times, and for the last 3 times, data were taken as the original data for analysis.

### 2.12. Sensory Evaluation

Sensory Evaluation was conducted according to GB/T 16291.1-2012 (the national standard for selection, training, and management of evaluators for sensory analysis is the standard of the People’s Republic of China). In the standard detailed requirements of sensory personnel selection, training, and sensory evaluation process and standards, the sensory evaluation part of this experiment was carried out in strict accordance with these requirements. The sensory evaluators were screened in advance as follows: Teachers and students of the National Marine Engineering Technology Research Center filled in the Preferred Evaluator Screening Questionnaire, from which qualified candidates were screened for a sensory ability using the screening training assessment. The results of various evaluations were combined, and 30 evaluators were finally selected to form the sensory evaluation team. The selection of sensory evaluators included the following aspects: Good sensory ability (basic taste, olfactory matching, three-point test, sequencing test) and the ability to sense, communicate, and describe volatile flavor substances of samples. The sensory evaluation was conducted with each sensory person providing heat-treated samples in separate coded trays. The different attributes were rated on a continuous sensory evaluation scale (Table 2) ranging from 0 (unperceived attributes) to 10 (maximum perceived intensity). If the degree of preference was lower than 5, it was unacceptable, and if it was equal to or higher than 5, it was acceptable. Acceptance criteria for other sensory indicators were: 1, 2, 9, and 10 were unacceptable; 3, 4, 5, 6, 7, and 8 were acceptable.

### 2.13. Statistical Analysis

Three parallel experiments were conducted on the same sample under the same conditions, and all experimental data were expressed as mean ± standard deviation. Data were analyzed using one-way ANOVA (comparison between mackerel mince products), Student’s *t*-test (comparison before and after heat treatment), and a correlation analysis using SPSS software (SPSS, version 16.0, Chicago, IL, USA), and a comparison of means was carried out using the Least Significance Difference (LSD) test and Duncan test. The level of significance was set at 95% (*p* < 0.05). Origin software (Origin, version 7.0, OriginLab, Northampton, MA, USA) was used to fit the formula.

## 3. Results

### 3.1. Measurements of Colour

**Figure 2:**
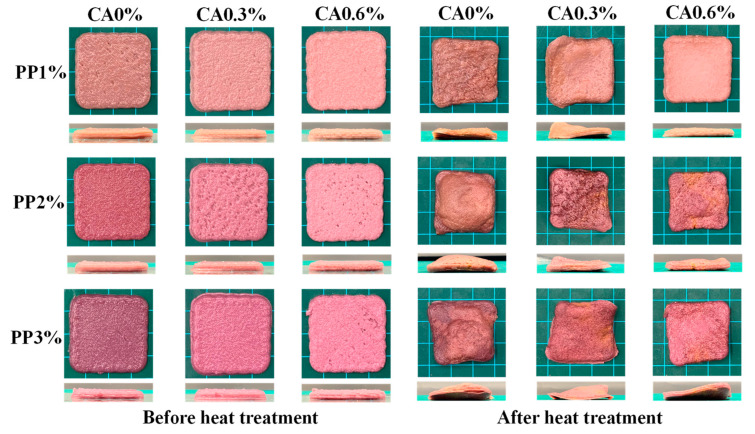
Printing quality of restructured mackerel mince.

Studies had shown that a bright colour such as red, orange, and yellow increase appetite, while black, brown, purple, and blue decrease appetite [21]. For the group without CA addition (Figure 2), the colour of the samples gradually changed from reddish-brown to dark red as the concentration of PP increased. PP contains anthocyanins, which are predominantly red in tone under acidic conditions and blue–green in tone under alkaline conditions [22]. In conjunction with the pH of the sample before heat treatment, it was found that the pH of the sample did not change significantly (about 6.0) with the increase in PP concentration when no CA was added. Therefore, the colour change of the sample was mainly caused by the concentration of PP. For groups with the same PP concentration and a different CA amount added, the samples gradually changed from purplish red to light pink with the increase in CA added. This was because, with the addition of CA, the pH of the samples decreased from 5.96~6.02 to 4.82~4.96, which in turn changed the colour of anthocyanins. After heat treatment, it was found that the overall colour of the product became darker, probably due to water loss and browning behavior of the sample. While the addition of CA inhibited the darkening of the samples after heat treatment, indicating that CA played a good role in colour protection, the greater the amount of CA added, the more significant its colour-protection effect. This was because during the processing, the balance of the redox reaction was broken, and under this condition, the CA reacted with oxygen first to protect the stain or pigment, so that the colour remained bright, thus inhibiting the browning of the mackerel mince [23].

**Figure 3:**
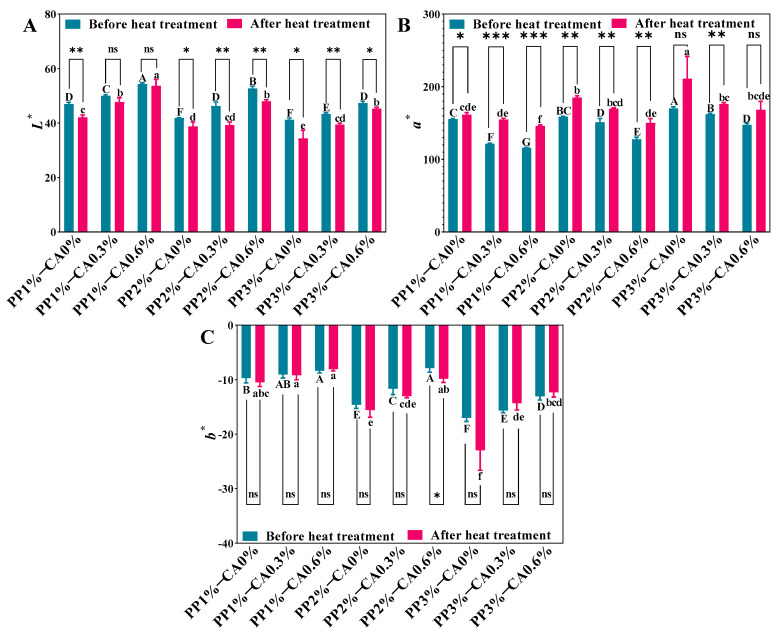
The colour of the restructured mackerel mince and its heat treatment product: (**A**−**C**) are the restructured mackerel mince’s L*, a*, b* of restructured mackerel mince before and after heat treatment. Different marked letters in each figure indicated statistically significant differences in data in the figure (*p* < 0.05); the letters “A−G” represent the significance between mackerel mince before heat treatment; the letters “a−f” indicate the significance between mackerel mince after heat treatment. The asterisks (*) indicate the significant differences between the two groups before and after heat treatment at *p* < 0.05; the asterisks (**) indicate the significant differences between the two groups before and after heat treatment at *p* < 0.01; the asterisks (***) indicate the significant differences between the two groups before and after heat treatment at *p* < 0.001; the mark (ns) indicates no significant difference between the two groups before and after heat treatment (*p* ≥ 0.05).

In order to verify the observed phenomenon and express the colour quantitatively, the change of colour in the 3D-printed sample before and after heat treatment were evaluated (Figure 3). Under the same CA addition, with the increase in PP concentration, the L* value decreased, a* value increased, and b* (negative) decreased, indicating that the increase in the concentration of PP made the raw and heat-treated products gradually dark and red, giving them attractive colour. For the group with the same concentration of PP, with the increase in CA added, the product’s L* value gradually increased, a* value gradually decreased, and b* value increased. The L* and b* values of these were higher, and a* value of these was lower, indicating that the colour of the product gradually became lighter, and the product gradually tended to be pink. This was because the anthocyanin contained in PP changes from purple to red due to the acidity, so with the increase in CA, it become acidic, and the colour gradually changed to pink [24]. Compared with raw samples, the L* value of heat-treated samples decreased and the a* value increased. Except for the decrease in the b* value of PP2%-CA0.6%, the b* value of other samples had no significant difference before and after heat treatment. This may be because of the high temperature of the sample due to microwave aging. The myoglobin in the mackerel mince was denatured. Wołoszyn et al. [25] found that the red (a*) intensity in the cooked meat was inversely proportional to the degree of denatured myoglobin. As a result, the heat-treated product had a stronger a* value. The L* values of the group with 0.6% CA were the highest, proving that they were more colourful, which was consistent with the visual observation of the samples (Figure 2). This suggested that the increase in PP concentration and CA amount could improve the colour of mackerel mince and give it an attractive appearance, and the CA could play a good role in protecting the colour of the samples during the heat-treatment process so that the samples still maintained a good colour after heat treatment. This was in line with the results of Li, Walker, and Faubion [26], who found that biscuits baked with CA in a convection oven retain the maximum anthocyanin values, preserving oxidation resistance and colour.

### 3.2. Rheological Properties of the Samples

Rheological properties of foods have a major impact on 3D printing by extrusion. Studies have shown that moderate viscosity and flow properties are necessary for food raw materials to be 3D printable [27]. To predict the extrusion effect through the nozzle, rheological properties such as yield stress (τ0), storage modulus (*G*′), and loss modulus (*G*″) are commonly used. τ0 and *G*′ are key indicators of the self-supporting ability of the matrix [28].

**Figure 4:**
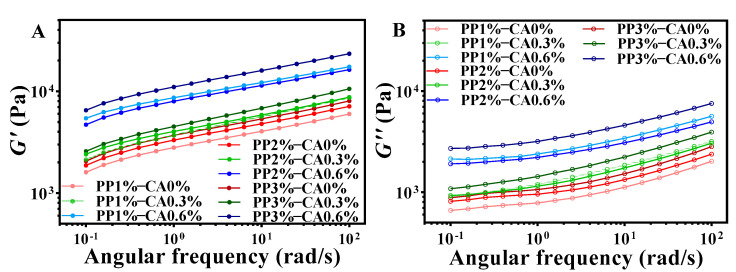
Frequency curves of restructured mackerel mince with different PP concentration and CA addition: (**A**) is the stored modulus (*G*′) of restructured mackerel mince; (**B**) is the loss modulus (*G*″) of restructured mackerel mince.

In this study, frequency scanning was used to evaluate the structural integrity and mechanical strength of the restructured mackerel mince. As shown in the Figure 4, the *G*′ of all the samples were higher than the *G*″ value, which indicated that the restructured mackerel mince was an elastic-based system with some rigidity and solid-like characteristics, which did not easily collapse between layers when the sample was deposited and formed in printing.

The addition of CA was able to increase the *G*′ and *G*″ and improve the apparent viscosity of the mackerel mince. This may be due to the addition of CA, combined with pH value; it can be seen that under the same PP concentration, the pH of mackerel mince decreased significantly with the increase in CA added, which will increase the charge repulsion on the starch network, change the structure, and produce more hydrophobic interactions. Furthermore, the addition of salt in the preparation of mackerel mince can mask the electrical charges on the proteins, thus potentially creating a stronger network structure [29]. Therefore, it may strengthen PP and enhance the overall viscoelasticity. The *G*′ and *G*″ values of the products increased with increasing the amount of CA added at the same PP concentration, and when the CA amount added reached 0.6%, both the *G*′ and *G*″ values of the samples were significantly higher than the remaining two additions, indicating that CA could improve the elasticity of the restructured mackerel mince. These were consistent with the results of He [9], who found that the addition of CA decreased the pH, resulting in the increase in *G*′ and the decrease in the fluidity of PP. Furthermore, the *G*′ and *G*″ values also increased gradually with the concentration of PP when CA was added at 0% and 0.3%, but the trend of increase was smaller in magnitude. This indicates that the increase in PP concentration could also improve the elasticity and reduced the fluidity of the restructured mackerel mince, but the effect was lower than that of CA.

**Figure 5:**
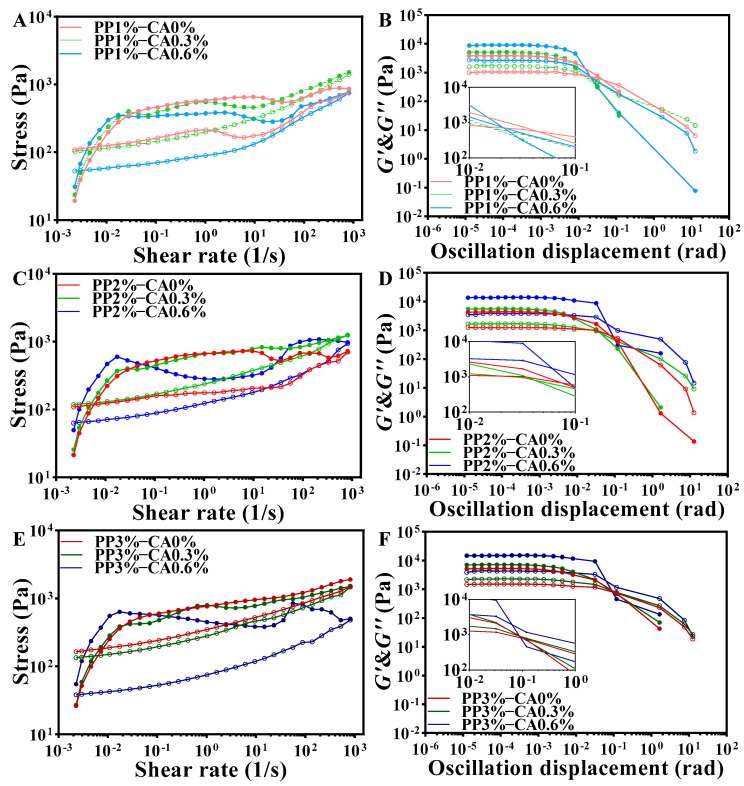
Rheological results of restructured mackerel mince with different PP concentration and CA amounts: The stored modulus (*G*′) and the loss modulus (*G*″) are represented by solid and hollow icons respectively; (**A**,**C**,**E**) are the thixotropic hysteresis loop-stress ramps of restructured mackerel mince; and (**B**,**D**,**F**) are the yield stresses of restructured mackerel mince.

Gels with thixotropic properties are more suitable for extruded 3D printing. The viscosity of the gel decreases during shearing, which facilitates flow control during extrusion; after printing, the gel rethickens and regains its structure after shear stress is removed [30]. Thixotropy was determined by the hysteresis curve area; the larger the hysteresis area, the stronger the thixotropic behavior. From the Figure 5, it could be observed that typical hysteresis curves, i.e., thixotropy, could be observed for all restructured mackerel mince. This might be because a moderate addition of PP and CA makes linked protein clusters non-expandable and fragile, and the bonds can break reversibly under stress, temporarily reducing viscosity but not storing much elastic energy [31]. In addition, the region of the hysteresis curve between the upper and lower curves is attributed to the degree of breakdown and alignment of the internal structure of the samples. The endpoint of the downward curve is higher than the beginning of the upward curve for all products, which indicates that these samples can be returned to their original state in a short time [17]. At the same PP concentration, the hysteresis area was larger when CA was added to 0.6% and the thixotropic behavior became stronger [32].

The yield stress (τ0) is defined as the intersection of the *G*′ and *G*″ [8], which is closely related to the extrusion performance of the ink during 3D printing, reflecting the mechanical strength of the mixed gel ink [33] and the minimum force required to initiate fluid flow [34]. For the same CA amount added, the yield stress of samples increased with the increase in PP concentration (Figure 5B,D,F). This was probably because PP absorbed water sufficiently and swelled up, and when its volume expanded to a certain limit, the particles broke off and dispersed in all directions. The expanded starch molecules could connect with each other to form a network of water-containing colloid particles. This makes the whole structure of the mackerel mince firmer and thus requires more force to push it out. Similarly, the yield stress increased with the addition of CA when the concentration of PP was the same. This could be because the increase in CA increased the viscoelasticity of mackerel mince, which meant that the printer’s extrusion device needed more extrusion to extrude the sample smoothly. This could be because after the addition of CA to PP, the esterification process begins between the hydroxyl groups that make up the amylose and amylopectin molecules and the carboxylic acid in the CA structure [35], and the presence of ester groups causes molecular repulsion and spatial hysteresis, which prevents the molecular interaction of amylose and leads to a strengthening of starch network [36].

In summary, the rheological results showed that the moderate increase in PP concentration and CA amount made the mackerel mince suitable for 3D-printing extrusion. However, the excessive addition of the two could lead to poor fluidity and increased yield stress of the mackerel mince, which makes the sample not conducive to 3D-printing extrusion.

### 3.3. 3D Printability

To evaluate the effect of PP concentration and CA content on the 3D-printing properties of mackerel mince, the visual pictures of the 3D-printed samples before and after microwave heat treatment were taken (Figure 2), and the 3D-printing accuracy was measured (Table 3). The smaller difference between the accuracy of the printed products and the print geometry parameters (42 mm for the side length and 4 mm for the height) represented a better printing effect, and the larger change in accuracy before and after heat treatment indicated a greater degree of deformation. The shape of all products after printing was similar to the size of the printed geometry. Among them, the printing accuracy of PP1%-CA0% was 99.68 ± 0.36%, which was closest to the standard geometry. This may be because the concentration of 1% PP increased the viscoelasticity of mackerel mince, giving the mackerel mince the right fluidity, so that the 3D printing of mackerel mince had a better extrusion effect and better printing precision. When the PP concentration was 1% and 2%, CA decreased the printing accuracy of mackerel mince. When PP concentration was 3%, 0.6%CA caused a significant reduction in printing accuracy. In addition, a loose structure, rough surface, and porosity were also observed in the samples with higher CA content. This was because the addition of CA reduced the fluidity of mackerel mince, and the printing was not smooth. With the same amount of CA added, the printing accuracy of different PP concentrations did not change significantly, so PP had little effect on the printing accuracy of mackerel mince. This may be due to the fact that CA and PP were dissolved in boiling water during the preparation process, and the same degree of starch esterification and cross-linking occurred for the same amount of CA and a smaller increase in PP [37]. Therefore, the effect of PP on the printing accuracy was less than that of CA.

After heat treatment, it could be seen from the Figure 2 that all 9 samples had varying degrees of deformation. When the concentration of PP was 3%, the increase of the added amount of CA had no significant changes in the side length and high deformation degree. When the concentration of PP was low, the addition of CA would cause side-length and height deformation, indicating that a small concentration of PP would shrink the whole sample. Combined with the 3D-printing diagram, it could be seen that the increase of PP concentration and CA addition could increase the deformation degree of the heat-treated product, and the deformation degree of the side length and height increases from 7.82 ± 1.01 and 5.73 ± 2.04% to 16.05 ± 2.41 and 18.98 ± 1.49%, respectively. Although the increase of the CA amount and PP concentration could reduce the 3D printability, the printing accuracy was still as high as 95.59~99.68%, which was still within the acceptable range [17]. Other substances could also be considered for improvement in the later stage.

### 3.4. Measurements of pH

**Figure 6:**
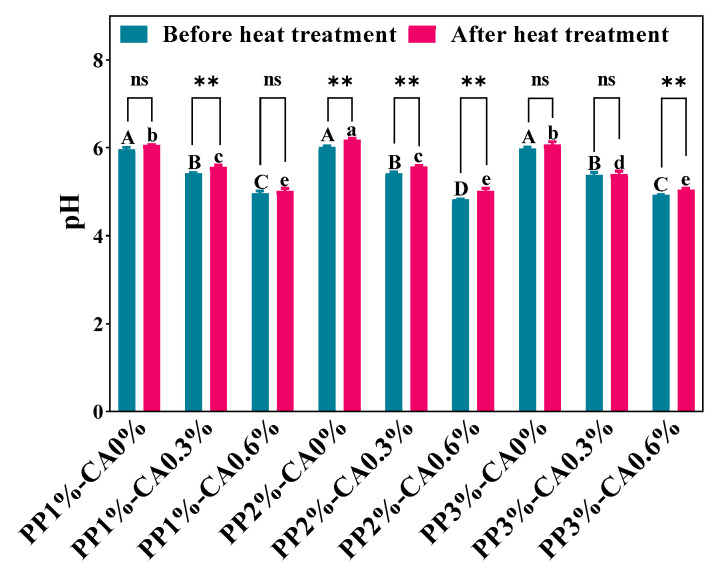
The pH of the mackerel mince before and after heat treatment. The letters “A–D” represent the significance between mackerel mince before heat treatment; the letters “a–e” indicate the significance between mackerel mince after heat treatment (*p* < 0.05). The asterisks (**) indicate the significant differences between the two groups before and after heat treatment at *p* < 0.01; the mark (ns) indicates no significant difference between two groups before and after heat treatment (*p* ≥ 0.05).

CA is acidic, and it will make the food acidic to some extent and can mask the bitterness and improve the palatability of the food [38]. However, inappropriate pH changes can alter protein solubility, rheological properties, and gel characteristics, thus affecting the quality of 3D printed mackerel mince products. As shown in Figure 6, the pH of the samples gradually decreased from about 6 to 4.82 with the increase in CA additions at the same PP concentration. This was because the pH value of CA was about 2–2.5, and with the increase in CA, the content of H+ in the whole system increased, which makes the pH value of the whole system decrease. For the group without CA, different PP concentrations did not significantly change the pH of the samples (*p* > 0.05), thus, the concentration of PP could not change the pH of the samples. Similarly, for the group with CA of 0.3%, the pH value between the different PP concentration groups were not significant (*p* > 0.05). After heat treatment, in the 2% PP concentration group, the pH values of PP1%-CA0.3% and PP3%-CA0.6% increased, and the pH changes after heat treatment showed the same trend as those before heat treatment.

In general, CA had a great influence on the pH of samples, while PP and the microwave treatments did not cause significant changes to pH.

### 3.5. Texture Properties

**Figure 7:**
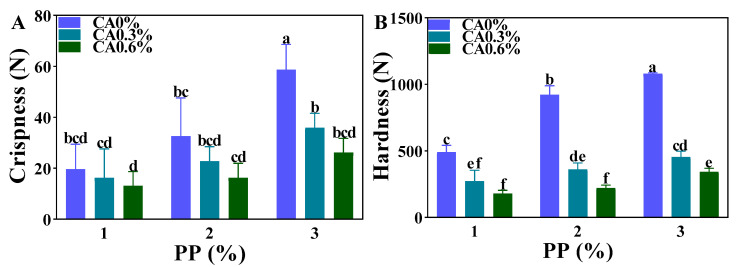
The texture of the heat-treated product: (**A**) is the crispness of heat-treated product; (**B**) is the hardness of heat-treated product; different marked letters in each figure indicated statistically significant differences in data in the figure (*p* < 0.05).

Hardness and crispness are important attributes used to evaluate the acceptability of microwave flake products [39]. For the sample without CA, the crispness of the product increased significantly when the concentration of PP was increased to 3% (Figure 7). As the concentration of PP increased, the starch content in mackerel mince increased, and the degree of polymerization of amylose in PP was large, such that the gel hardened after gelatinization [40], resulting in increased crispness and hardness. For the low CA content group (0% and 0.3%), the crispness of the product increased with the increase in the PP concentration, but for the high CA content group (0.6%), the change in the PP concentration did not significantly affect the crispness of the sample. This could be because with using microwave, the high concentration of CA resulted in a severe esterification reaction with PP [41], which damaged the surface of PP and changed its properties, leading to the reduction of crispness and hardness. The addition of CA had a greater effect on the hardness of the product, resulting in a substantial decrease in hardness. In conclusion, the increase in PP and CA concentrations changed the texture characteristics of heat-treatment products. PP enhanced the hardness and crispness of mackerel mince, while CA played the opposite role. Therefore, products with different crispness and hardness were obtained by adjusting the ratio of the two.

### 3.6. Measurements of LF-NMR

The LF-NMR can obtain information about the state, distribution, and migration of water in food by measuring the relaxation time of hydrogen protons. Bound water, free water, and immobile water are the three major water groups in food systems, and the transverse relaxation time *T*_2_ is usually selected as the basis for the classification of different water groups, which is related to the binding force and degree of freedom of hydrogen protons and reflects the chemical environment of hydrogen protons in the samples. Generally speaking, the shorter *T*_2_ relaxation time indicates that the water molecules are more tightly bound to the macromolecules and that the water is less mobile [42].

**Figure 8:**
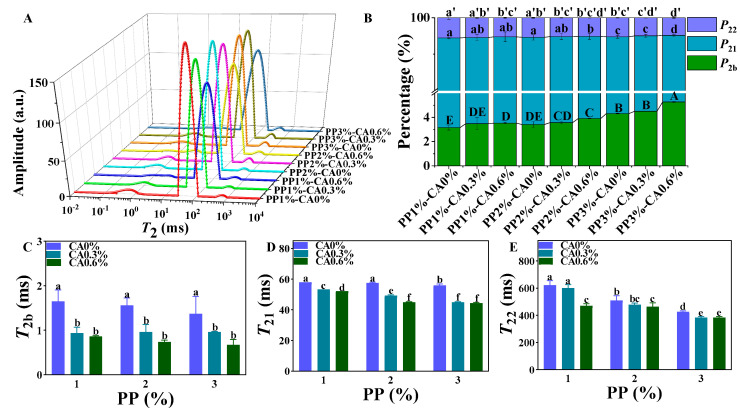
LF-NMR results of the restructured mackerel mince 3D-printing product before heat treatment: (**A**) is moisture distribution of the restructured mackerel mince 3D-printing product, (**B**) is the *P*_2b_, *P*_21_, and *P*_22_ of the restructured mackerel mince 3D-printing product (A–E, a–d, and a’–d’ represent the significance of *P*_2b_, *P*_21_, and *P*_22_, respectively), and (**C**–**E**) are the *T*_2b_, *T*_21_, and *T*_22_ of the restructured mackerel mince 3D printing product; different marked letters in each figure indicate statistically significant differences in data in the figure (*p* < 0.05).

Three characteristic peaks (Figure 8) appeared in the LF-NMR relaxation time (*T*_2_) curve of fish minced samples, which represent bound water (*T*_2b_), immobilized water (*T*_21_), and free water (*T*_22_) [43]. The *T*_21_ was the main peak among the three peaks, with a relative content of 94.24~96.40%, indicating that the water in the restructured mackerel mince exists mainly in the state of immobilized water after 3D printing (Figure 8A).

With the same concentration of PP and increasing CA amounts, the *T*_2b_, *T*_21_, and *T*_22_ relaxation times migrated to a shorter relaxation time, while the relative content of *P*_2b_ increased and that of *P*_21_ and *P*_22_ decreased. The mackerel mince with a PP concentration also showed the same trend with the addition of CA. This indicated that the increase in PP and CA could enhance the stability and ability of the combined water in mackerel mince and reduce the fluidity of water in mackerel mince. This was because the starch gelatinizes with increasing PP and binds more water to form a network of aqueous colloids. With the increase in CA content, the enhanced water-binding ability observed may be due to the increase in carboxyl groups, which dehydrate to produce anhydride, and the anhydride reacts with PP starch molecules for esterification, interrupting the linear structure of amylose and branched structure of amylopectin [44]. The steric-hindrance effect weakens the interaction between starch molecules. Furthermore, the free water was promoted to penetrate into the amorphous area of starch through chemical forces and be converted into bound water, and the free water absorbed by PP molecules was also converted into bound water, thus improving the overall water-binding ability of mackerel mince [45]. The results showed that a moderate increase in PP concentration and CA addition could enhance the binding ability of water molecules to a certain extent, thus reducing the fluidity of water in the sample and improving the 3D-printing effect of mackerel mince. However, if too much PP concentration and CA was added, the fluidity of mackerel mince would be reduced, which would make the 3D printing of mackerel mince not smooth.

### 3.7. Measurements of E-Tongue

**Figure 9:**
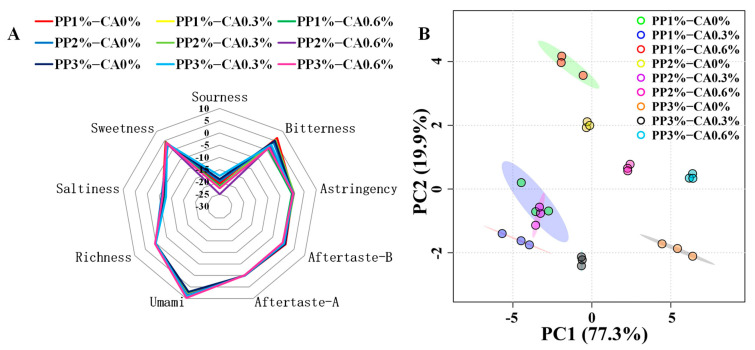
E-tongue result of the heat-treatment product: (**A**) is the E-tongue radar chart of the heat-treated product, and (**B**) is the PCA analysis of heat-treatment products.

As a rapid detection technique in food processing that satisfies consumers’ quests for nutrition and flavor, this test is based on the construction of a mathematical model describing and simulating human taste system [46]. Richness refers to the aftertaste, which reflects the persistence of the taste of the sample. Aftertaste-B reflects the degree of residual bitterness, and aftertaste-A reflects the degree of residual astringency. Potassium chloride (KCl) and tartaric acid were used to formulate the reference solution, and the taste value was the tasteless point, which was measured as −13 for the sour taste, −6 for the salty taste, and 0 for the rest of the tastes, as a benchmark; when the taste value of the sample is lower than the tasteless point, it means that the sample does not have taste and vice versa. It could be seen from Figure 9 that the sour and salty taste values of the products were lower than the tasteless point. The astringency, aftertaste, and richness values of these aquatic products were close to the odorless point, and other taste indicators were effective taste indicators. The sweetness and bitterness aftertaste taste values of all products were more concentrated with no basic differences. With the increase in PP concentration and CA addition, the bitterness gradually weakened and the umami value gradually increased. The reason was that PP and CA had sweet and sour taste respectively. With the increase of the PP concentration and CA added, the sweet and sour tastes gradually grew stronger, which effectively inhibited the bitter taste. However, the sour taste basically had no inhibiting effect on the sweet taste [47], and the sweet had a soothing effect on the sour taste. Umami is a signal of protein. PP was rich in protein, so with the increase in PP concentration, the protein content in the overall mackerel mince was gradually enriched, and the amino acid content of umami gradually increased, so the umami flavor of mackerel mince gradually increased.

Principal component analysis (PCA) is commonly used to extract information from variables that primarily affect the spatial distribution of samples. This process allows for reducing the dimensionality of the data matrix while retaining most of the information from the original data and explaining the relationships between objects and the correlation structure of the variables [48]. Therefore, PCA analysis was performed on the response value dataset of e-tongue (Figure 9B) to assess the effect of the increase in PP concentration and CA content on the taste of mackerel mince. The PC1 and PC2 accounted for 77.3% and 19.9% of the total variance, respectively, and the cumulative contribution of the first two PCs accounted for 97.2%, indicating that they were sufficient to explain the total variance in the dataset [49]. It could be observed that in the PCA analysis graph, it was easy to distinguish the samples with different concentrations and amounts of PP and CA; the products with 0.6% CA were located in the positive half-axis of y-axis, the products with 0.3% CA were located in the negative half-axis of x-axis. The smaller the distance between samples, the smaller the difference. Therefore, under the same CA amounts, samples with different PP concentration were in the same area, but there was still a certain distance and no coincidence between them, indicating that there were some differences.

In conclusion, the PP concentration and CA amount added had an effect on the taste of mackerel mince, which could produce different tastes at different concentrations and additions and could be effectively differentiated.

### 3.8. Sensory Analysis

After a comprehensive instrumental sensory evaluation, descriptive sensory evaluation was performed for the texture, flavor, and taste of the products after heat treatment. It could be seen from Table 4 that for the same PP concentration groups, the colour score decreased with the increase in CA additions, which may be because the pH of the sample gradually decreased with the increase in CA additions, making the colour of anthocyanin gradually change from dark red to light pink. These were consistent with the results obtained from our observation and testing with the colour-difference meter. At the same PP concentration, the more CA added, the lower the hardness and crispness scores of heat-treated products, which were considered to be softer. When the amount of CA was constant, the hardness and crispness increased with the increase in PP concentration. This may be because under microwave, with the increase in PP concentration, the degree of polymerization of amylose was larger, the gel hardened after gelatinization, and the crispness and hardness increased [40]. However, the significant difference of sensory evaluation results was not obvious compared with the instrumental measurement, which indicated that sensory evaluators believed that the samples had similar hardness and brittleness. With the increase of CA, PP gelatinizes to form an irregular and porous network structure. CA reacted with the surface molecules of PP to penetrate into the interior and fully react with PP, which changed its properties and lead to the decrease in crispness and hardness [41]. This was consistent with the texture properties. The different tastes were scored by tasting and smelling; by taste, the fishy and sweet tastes were perceived by sensory personnel as becoming progressively lighter with increasing CA amounts at the same PP concentration, likely due to both of them being affected by the sourness brought about by CA, which increased with CA additions. Under the condition of the same CA amount, the increase in PP concentration faded the fishy taste but enhanced the sweetness of mackerel mince, which could be due to the fact that PP contains sugar, thus enhancing the overall sweetness of mackerel mince and gradually weakening the sour taste. The gradual increase in PP and CA concentrations made the savory, fresh, and aftertaste profiles of the mackerel mince stronger due to PP and CA bringing about more abundant protein and sweet and sour tastes, which increased the taste of the mince itself to stimulate the taste buds and enhanced the aftertaste and umami taste of the mince. This indicated that the increase in PP concentration and CA addition could enrich the taste of mackerel mince and weaken its fishy, sour, and other undesirable tastes. The scores of the above indicators were within 3~8 points, which was within the acceptable range. At the same PP concentration, with the increase in CA additions, the sensory personnel’s degree of preference for the product showed a trend of first increasing and then decreasing, and the degree of preference score of 0.6%CA was higher than that of the samples without CA addition. When the amount of CA was the same, the degree of preference score obtained by increasing the concentration of PP had the same trend. According to the acceptable range stipulated by the sensory evaluation, except for PP1%-CA0%, the degree of preference of other products was above 5 points, which was acceptable to the public. Among them, PP2%-CA0.3% had the highest degree of preference score and was the most popular.

### 3.9. Correlation Analysis

Based on the results of the previous experiments on the regulation of mackerel mince by PP and CA, the correlation analysis was performed on the pH, rheological properties, moisture distribution properties, and 3D printing accuracy; the results are shown in Table 5. Table 5 showed that there was a correlation between all these variables. There was a high positive correlation between printing accuracy and *T*_2b_ and pH of the mackerel mince, further indicating that the printing accuracy of the mackerel mince was improved by improving *T*_2b_ and pH, thus indicating that the changes in *T*_2b_ and pH of the mackerel mince occurred simultaneously with the increase of PP concentration and CA addition and mutually promoted each other. The change in the LF-NMR of the mackerel mince led to a change in the internal moisture of the mackerel mince, which further improved the printing accuracy of the mackerel mince. Therefore, the pH and *T*_2b_ index of mackerel mince could be used as an important reference for measuring the 3D printing of mackerel mince.

**Figure 10:**
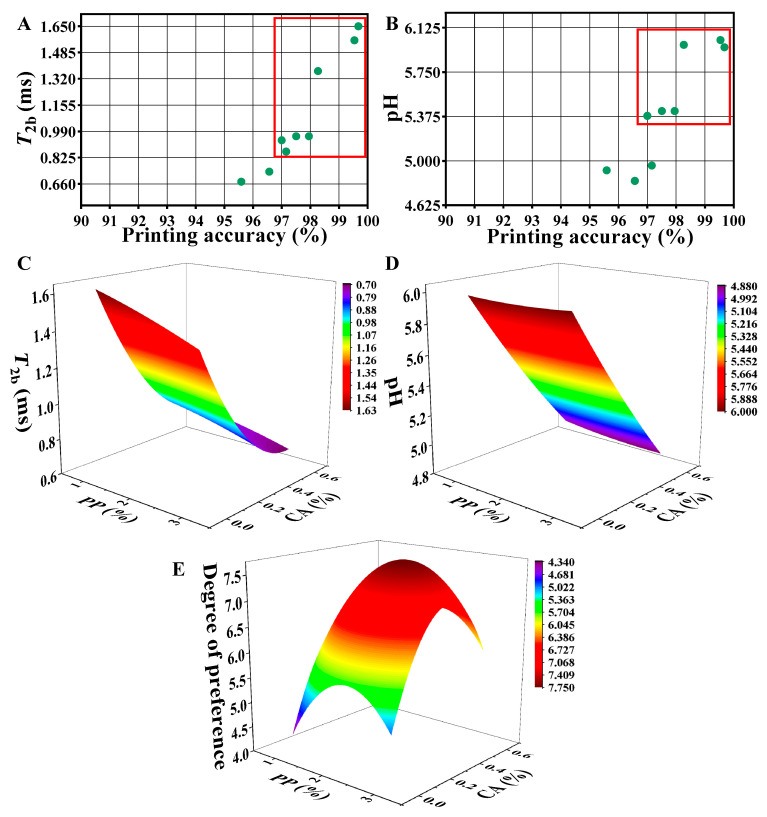
Correlation analysis data charts: (**A**,**B**) are the effects of *T*_2b_ and pH on the accuracy of 3D printing; (**C**–**E**) are the nonlinear surface fitting of *T*_2b_, pH, and degree of preference to PP and CA, respectively. (**A**,**B**) use red box coils to print points with an accuracy greater than 97%.

It could be seen from Figure 10 that when the *T*_2b_ and pH values of the previous experiments were analyzed with the increase in PP and CA concentrations and the printing accuracy, it was clear that when the printing accuracy reached 97%, the corresponding pH was between 5.38~6.13 and the corresponding *T*_2b_ value was between 0.83~1.65 ms. In order to meet the high degree of printing accuracy, the range of pH should be controlled between 5.38~6.13, as the most suitable pH, and the range of *T*_2b_ value should be controlled between 0.83~1.65 ms as the most suitable *T*_2b_ value.

Secondly, sensory evaluation was the standard used to determine the quality of the heat-treatment products in this study. Among the indexes of sensory evaluation, the scores of other indexes, except degree of preference, were all within the acceptable range. Therefore, the degree of preference was taken as the limit of sensory evaluation required to fit with the PP and CA concentrations. The acceptable range of the degree of preference was 5~10 points.

The pH, *T*_2b_, and degree of preference (DP) values were fitted to a nonlinear surface with PP concentration (*x*) and CA addition (*y*) to obtain Equations (3)–(5).

(3) $$\mathrm{pH}=6.02-0.05x-1.96y+0.01x^{2}+0.43y^{2}-0.05\mathrm{xy},$$

(4) $$T_{2b}=1.70-0.06x-2.71y-0.01x^{2}+2.13y^{2}+0.08\mathrm{xy},$$

(5) $$\mathrm{DP}=1.10+4.22x+12.14y-0.98x^{2}-17.37y^{2}-0.04\mathrm{xy},$$

Under the condition that the ranges of pH, *T*_2b_, and DP values were determined to be 5.38~6.13, 0.83~1.65 ms, and 5~10, respectively; the corresponding concentrations of PP and CA were obtained by calculating the ranges of 1.00~3.00% and 0.09~0.32%, respectively.

**Figure 11:**
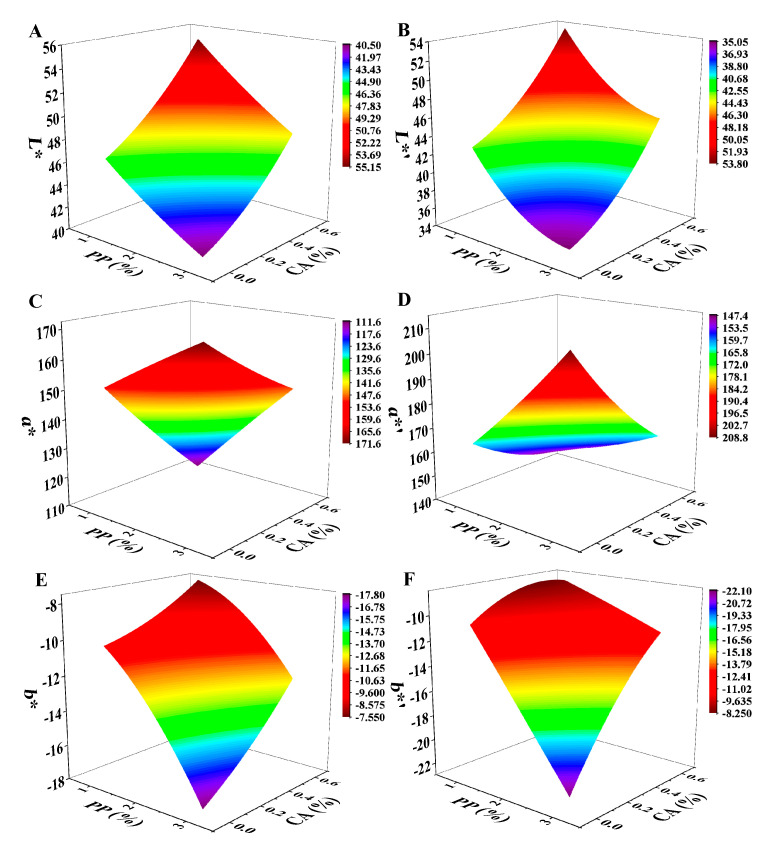
Correlation analysis data charts: (**A−F**) are the nonlinear surface fitting of L*, a*, b*, L*′, a*′, and b*′ to PP and CA, respectively. L*, a*, and b* are the colour indexes of mackerel mince before heat treatment and L*′, a*′, and b*′ are the colour indexes of mackerel mince after heat treatment.

The L*, a*, and b* values were fitted to the nonlinear surface with the concentrations of PP (*x*) and CA (*y*) used to obtain Equations (6)–(11) (Figure 11).

(6) $$L*=50.20-4.13x+9.78y+0.30x^{2}+9.69y^{2}-1.05\mathrm{xy},$$

(7) $$a*=139.84+11.63x-86.38y-0.36x^{2}+11.10y^{2}+13.95\mathrm{xy},$$

(8) $$b*=-8.84-0.66x-0.09y-0.78x^{2}+3.99y^{2}+2.19\mathrm{xy},$$

(9) $$L*\prime =52.01-10.88x+8.33y+1.75x^{2}+17.30y^{2}-0.55\mathrm{xy},$$

(10) $$a*\prime =145.65+16.84x-27.90y+1.40x^{2}+40.81y^{2}-23.84\mathrm{xy},$$

(11) $$b*\prime =-5.17-5.44x+3.72y-0.06x^{2}-11.68y^{2}+6.86\mathrm{xy},$$

Substituting the ranges of PP concentration and CA addition, the ranges of L*, a*, and b* before heat treatment were obtained as 43.62~47.23, 144.68~158.38, and −15.36~−10.06, respectively, and the ranges of L*, a*, and b* after heat treatment were 39.03~43.72, 159.56~181.13, and −15.45~−9.81, respectively.

This showed that by controlling the range of printing accuracy (>97%) and the acceptable range of degree of preference (>5), the optimal PP concentration was 1.00~3.00% and the optimal CA addition was 0.09~0.32%. Hence, that the L*, a*, and b* of restructured mackerel mince could be regulated from 43.62 to 47.23, from 144.68 to 158.38, and from –15.36 to −10.06, and heat-treatment products could be regulated from 39.03 to 43.72, from 159.56 to 181.13, and from −15.45 to −9.81, respectively, and the range of degree of preference was 5.29~7.01. Therefore, through 3D-printing accuracy and the degree of preference, the optimal PP and CA concentrations could be obtained, thus realizing the colour regulation of mackerel mince.

## 4. Conclusions

In this study, the PP concentration and CA amount increased in the mackerel mince for restructuring, which realized the colour regulation of mackerel mince, which in turn had a high printing accuracy in a certain range. The results showed that the increase in the PP concentration increased the a* value of mackerel mince and decreased the L* value of the mackerel mince. Conversely, the increase in CA addition increased the L* value but decreased the a* value of mackerel mince, which made the product colour gradually brighter and pink. After heat treatment, the L* value decreased and a* value increased. Except for the decrease in the b* value of PP2%-CA0.6%, the b* value of other samples had no significant difference before and after heat treatment. The increase in PP concentration and CA addition increased the water-binding capacity of mackerel mince and reduced the binding ability of water molecules. Appropriate PP and CA concentrations moderately increased the *G*′, *G*″, and yield stress of mackerel mince, making it suitable for 3D printing. Meanwhile, the increase in PP concentration and CA addition enhanced the umami and sweet taste of mackerel mince but reduced the fishy and sour taste. Except PP1%-CA0%, the degrees of preference of other samples were within the acceptable range, and when the concentration of PP was 2% and the amount of CA was 0.3%, it was welcomed by sensory personnel. When the printing accuracy range (>97%) and the acceptable range of degree of preference (>5) were controlled, the optimal PP concentration and CA addition were 1.00~3.00% and 0.04~0.40%, respectively. Thus, the colour of the mackerel mince and the heat-treated products could be adjusted, respectively. The results provide a new idea and reference for the development of mackerel leisure products. Based on this study, enterprises can develop new mackerel products to meet the special nutrition needs and tastes of consumers in the future.

## Figures and Tables

**Table 1:** Different restructured mackerel mince ratios.

Sample	PP (%)	CA (%)
PP1%-CA0%	1	0
PP1%-CA0.3%	1	0.3
PP1%-CA0.6%	1	0.6
PP2%-CA0%	2	0
PP2%-CA0.3%	2	0.3
PP2%-CA0.6%	2	0.6
PP3%-CA0%	3	0
PP3%-CA0.3%	3	0.3
PP3%-CA0.6%	3	0.6

PPx% in the table represents the concentration of purple potato powder, and CAy% represents the amount of citric acid added.

**Table 2:** Sensory evaluation form.

NAME	DATE
Samples Evaluated	Scoring Criteria
Heat treatment samples	Observe the heat-treated sample and the intensity of the colour, where the colour pink refers to too light and the colour purple refers to too dark (0 points for too light colour, 10 points for too dark colour).
	Taste heat-treated samples and evaluate the strength of hardness, where too weak means no need to use too much force when chewing and too strong means chewing with more force (0 points for too-weak hardness, 10 points for too-strong hardness).
	Touch and taste the strength of the crispness of the heat-treated sample. Too weak means the sample is not easy to break (soft texture during chewing) and too strong means the sample is fragile (easy to break during chewing). Please note that if you need to break a sample for brittleness sensory evaluation, please break the tasting sample and do not break the thermal sample for observation (0 points for too weak crispness, 10 points for too strong crispness).
	Smell and taste the heat-treated sample. The fishy smell is too strong if you think it is too obvious and too weak if it is too low (0 points for too weak fishy smell, 10 points for too strong fishy smell).
	Smell and taste the heat-treated sample. The umami is too strong if you think it is too obvious and too weak if it is too low (Umami flavor is too weak for 0 points, Umami flavor is too strong for 10 points).
	Smell and taste the heat-treated sample. The sweetness is too strong if you think it is too obvious and too weak if it is too low (0 points for too weak sweetness, 10 points for too strong sweetness).
	Smell and taste the heat-treated sample. The saltiness is too strong if you think it is too obvious and too weak if it is too low (0 points for too weak saltiness, 10 points for too strong saltiness).
	Smell and taste the heat-treated sample. The sour taste is too strong if you think it is too obvious and too weak if it is too low (0 points for too weak sour taste, 10 points for too strong sour taste).
	Smell and taste the heat-treated sample. The aftertaste is too strong if you think it is too obvious and too weak if it is too low (0 points for too weak aftertaste, 10 points for too strong aftertaste).
	Observe, touch, smell, and taste the heat-treated samples to evaluate the degree of preference of the samples (0 points for least preferred, 10 points for most preferred).

**Table 3:** Printing accuracy of restructured mackerel mince.

Sample	3D Printed Products/mm			Heat-Treated Products/mm		Degree of Deformation/%	
	Side Length	Height	Printing Accuracy/%	Side Length	Height	Side Length	Height
PP1%-CA0%	41.87 ± 0.15 ^b^	4.04 ± 0.14 ^a^	99.68 ± 0.26 ^a^	38.59 ± 0.55 ^a^	3.81 ± 0.05 ^a^	7.82 ± 1.01 ^c^	5.73 ± 2.04 ^b^
PP1%-CA0.3%	41.14 ± 0.21 ^c^	3.93 ± 0.03 ^ab^	97.95 ± 0.50 ^b^	37.70 ± 0.67 ^ab^	3.27 ± 0.08 ^d^	8.36 ± 1.25 ^c^	16.94 ± 2.69 ^a^
PP1%-CA0.6%	43.19 ± 0.62 ^a^	3.64 ± 0.02 ^c^	97.16 ± 1.49 ^bc^	37.07 ± 0.14 ^bc^	3.00 ± 0.78 ^e^	14.17 ± 1.08 ^a^	17.58 ± 0.87 ^a^
PP2%-CA0%	41.91 ± 0.22 ^b^	4.03 ± 0.01 ^a^	99.54 ± 0.18 ^a^	37.65 ± 1.00 ^ab^	3.65 ± 0.06 ^b^	10.16 ± 2.38 ^bc^	9.44 ± 1.71 ^b^
PP2%-CA0.3%	40.95 ± 0.31 ^c^	3.93 ± 0.06 ^ab^	97.51 ± 0.74 ^bc^	35.55 ± 0.26 ^d^	3.23 ± 0.11 ^d^	13.18 ± 1.10 ^ab^	17.84 ± 1.77 ^a^
PP2%-CA0.6%	43.44 ± 0.30 ^a^	3.61 ± 0.08 ^c^	96.57 ± 0.72 ^cd^	36.93 ± 0.86 ^bc^	2.95 ± 0.12 ^e^	14.98 ± 1.94 ^a^	18.21 ± 4.24 ^a^
PP3%-CA0%	42.21 ± 0.86 ^b^	4.01 ± 0.06 ^a^	98.27 ± 0.18 ^b^	35.87 ± 0.61 ^cd^	3.42 ± 0.07 ^c^	14.97 ± 2.97 ^a^	14.63 ± 2.13 ^a^
PP3%-CA0.3%	43.26 ± 0.29 ^a^	3.86 ± 0.05 ^b^	97.00 ± 0.70 ^bc^	36.51 ± 0.29 ^bcd^	3.16 ± 0.05 ^d^	15.61 ± 0.16 ^a^	18.12 ± 2.15 ^a^
PP3%-CA0.6%	43.85 ± 0.25 ^a^	3.55 ± 0.06 ^c^	95.59 ± 0.59 ^d^	36.82 ± 1.27 ^bcd^	2.87 ± 0.06 ^e^	16.05 ± 2.41 ^a^	18.98 ± 1.49 ^a^

Different superscript letters in the column indicate significant difference (*p* < 0.05).

**Table 4:** Analysis table of sensory evaluation results.

	Colour	Hardness	Crispness	Fishy Taste	Umami	Sweetness	Saltiness	Sour Taste	Aftertaste	Degree of Preference
PP1%-CA0%	6.56 ± 0.11 ^c^	5.72 ± 0.11 ^abc^	5.69 ± 0.11 ^a^	5.22 ± 0.02 ^a^	4.29 ± 0.11 ^e^	3.61 ± 0.51 ^cd^	4.44 ± 0.26 ^c^	4.00 ± 0.17 ^c^	4.51 ± 0.26 ^b^	4.56 ± 0.07 ^h^
PP1%-CA0.3%	4.39 ± 0.05 ^f^	5.48 ± 0.35 ^cd^	5.11 ± 0.10 ^c^	5.06 ± 0.04 ^abc^	4.61 ± 0.20 ^de^	3.56 ± 0.11 ^cde^	5.00 ± 0.15 ^ab^	4.39 ± 0.02 ^b^	4.53 ± 0.10 ^b^	6.33 ± 0.08 ^d^
PP1%-CA0.6%	3.56 ± 0.10 ^h^	4.67 ± 0.10 ^e^	4.72 ± 0.03 ^d^	4.87 ± 0.02 ^cd^	4.88 ± 0.02 ^cd^	3.22 ± 0.26 ^e^	5.06 ± 0.82 ^ab^	4.78 ± 0.17 ^a^	4.56 ± 0.10 ^b^	5.22 ± 0.04 ^f^
PP2%-CA0%	7.22 ± 0.18 ^b^	5.78 ± 0.01 ^ab^	5.78 ± 0.10 ^a^	5.17 ± 0.17 ^bc^	4.28 ± 0.11 ^e^	4.52 ± 0.05 ^ab^	4.89 ± 0.02 ^bc^	4.06 ± 0.03 ^c^	4.81 ± 0.75 ^ab^	5.33 ± 0.12 ^f^
PP2%-CA0.3%	5.11 ± 0.05 ^e^	5.61 ± 0.01 ^bcd^	5.17 ± 0.09 ^c^	4.99 ± 0.11 ^bcd^	5.00 ± 0.80 ^cd^	4.44 ± 0.28 ^b^	5.11 ± 0.01 ^ab^	4.11 ± 0.02 ^c^	4.82 ± 0.11 ^ab^	7.61 ± 0.05 ^a^
PP2%-CA0.6%	4.17 ± 0.05 ^g^	5.39 ± 0.01 ^d^	5.06 ± 0.26 ^c^	4.78 ± 0.17 ^d^	5.67 ± 0.02 ^ab^	3.44 ± 0.20 ^de^	5.39 ± 0.02 ^ab^	4.58 ± 0.03 ^b^	4.83 ± 0.02 ^ab^	7.00 ± 0.01 ^c^
PP3%-CA0%	8.22 ± 0.02 ^a^	6.00 ± 0.15 ^a^	5.87 ± 0.11 ^a^	5.17 ± 0.11 ^bc^	4.83 ± 0.26 ^cd^	4.94 ± 0.10 ^a^	5.11 ± 0.02 ^ab^	3.72 ± 0.05 ^d^	4.96 ± 0.02 ^ab^	5.06 ± 0.07 ^g^
PP3%-CA0.3%	6.22 ± 0.06 ^d^	5.72 ± 0.17 ^abc^	5.44 ± 0.11 ^b^	4.94 ± 0.17 ^cd^	5.17 ± 0.01 ^bc^	4.78 ± 0.17 ^ab^	5.17 ± 0.11 ^ab^	4.11 ± 0.07 ^c^	5.00 ± 0.01 ^ab^	7.17 ± 0.04 ^b^
PP3%-CA0.6%	5.00 ± 0.22 ^e^	5.44 ± 0.17 ^cd^	5.24 ± 0.04 ^bc^	4.28 ± 0.08 ^e^	5.71 ± 0.01 ^a^	3.89 ± 0.02 ^c^	5.56 ± 0.05 ^a^	4.46 ± 0.17 ^b^	5.33 ± 0.26 ^a^	5.67 ± 0.09 ^e^

Different superscript letters in the column indicate significant difference (*p* < 0.05).

**Table 5:** Correlation between variables.

Indicator	Printing Accuracy	pH	*G*′	*G*″	τ0	*P*_2b_	*P*_21_	*P*_22_	*T*_2b_	*T*_21_	*T*_22_
Printing accuracy	1										
pH	0.89 **	1									
*G*′	−0.86 **	−0.86 **	1								
*G*″	−0.89 **	−0.86 **	0.99 **	1							
τ0	−0.81 **	−0.72 *	0.82 **	0.80 **	1						
*P*_2b_	−0.74 *	−0.40	0.64	0.69 *	0.72 *	1					
*P*_21_	0.73 *	0.39	−0.64	−0.68 *	−0.72 *	−1.00 **	1				
*P*_22_	0.89 **	0.63	−0.72 *	−0.76 *	−0.76 *	−0.88 **	0.87 **	1			
*T*_2b_	0.95 **	0.94 **	−0.79 *	−0.81 **	−0.70 *	−0.53	0.52	0.74 *	1		
*T*_21_	0.92 **	0.82 **	−0.70 *	−0.73 *	−0.79 *	−0.67 *	0.66	0.88 **	0.88 **	1	
*T*_22_	0.70 *	0.41	−0.57	−0.60	−0.56	−0.83 **	0.83 **	0.91 **	0.52	0.67 *	1

*: *p* < 0.05; **: *p* < 0.01.

## Data Availability

The data presented in this study are available upon reasonable request.
